# Conventional Processing Affects Nutritional and Antinutritional Components and *In Vitro* Protein Digestibility in Kabau (*Archidendron bubalinum*)

**DOI:** 10.1155/2021/3057805

**Published:** 2021-01-23

**Authors:** Aprilia Fitriani, Umar Santoso, Supriyadi Supriyadi

**Affiliations:** ^1^Department of Food Technology and Agricultural Products, Faculty of Agricultural Technology, Universitas Gadjah Mada, Flora Street 1, Yogyakarta 55281, Indonesia; ^2^Food Technology, Faculty of Life Science, Surya University, M.H. Thamrin Street KM 2.7 Grand Serpong Mall 1st Floor F8 and F9 Units, North Panunggangan, Pinang, Tangerang City, Banten 15143, Indonesia

## Abstract

Kabau, an unexplored crop, was analyzed to determine its nutrition and antinutrition components and *in vitro* protein digestibility (IVPD). Some conventional processes, such as steaming, frying, and boiling, were carried out to study their effect. The results indicated that all of the techniques reduced carbohydrate content. Frying significantly increased the fat content of Kabau and reduced other nutritional components. In general, all the methods significantly reduced phytic acid, tannin, and trypsin inhibitors, as much as 94.95–96.26%, 20–35%, and 89.22–92.88%, respectively. The reduction of antinutritional components resulted in higher IVPD on boiled and steamed Kabau, 69.47% and 61.48%, respectively.

## 1. Introduction

Kabau peas belong to the Fabaceae family, mostly found in tropical regions such as Indonesia, Thailand, Malaysia, and the Philippines [1]. Kabau is categorized as an indigenous Indonesian plant and is commonly found in Sumatra and Kalimantan. Kabau is known by different names depending on each region. It is known as Bangkong in Java; Kabau and Jengkol Utan in Sumatra; Jiring Tupai, Kabau, and Saga Gajah in Malaysia; and Bugas in the Philippines [2, 3]. Besides, Malaysia also knows Kabau as Kerdas and Nieng-NOK in Thailand [4]. Kabau has a foul odor and taste like Jengkol (*Pithecellobium jiringa*) or Petai (*Parkia speciosa*). The consumption of Kabau usually leads to smelly stools, sweat, urine, or even mouth odor [5].

Kabau is rich in protein, carbohydrate, and low fat. Crude protein, carbohydrate, and fat of matured Kabau are 17.2, 78.5, and 0.46 g/100 g dry weight, respectively. Kabau has 386.8 kcal/100 g energy [5]. The protein content indicates that Kabau could be used as an alternative protein source. However, it is also stated that Kabau contains a significant amount of antinutritional components. These antinutritional components include trypsin inhibitors [1, 5, 6], tannin, and hemagglutinin [5]. Tannin, trypsin inhibitor, and hemagglutinin of Kabau are 0.46 g/100 g wet weight, 12.5 TIU/ml, and 1280 HU/g wet weight, respectively [5].

Kabau is commonly consumed in fresh form. Its antinutritional components can hinder the digestibility of its protein if it was eaten without processing. The habit of consuming Kabau in its raw form causes the body to not fully absorb the Kabau protein. The antinutritional components of some seeds/legumes/peas can be reduced by processing, especially heat treatment [2, 7–9].

Studies on the effects of processing on nutritional and antinutritional components have indeed been found in cereals and nuts [7–11]. Extrusion (148°C) of pea seeds (*Pisum sativum*) did not change protein content but significantly reduce some antinutritional components such as phytic acid, tannins, trypsin, chymotrypsin, and *α* amylase inhibitors and hemagglutinin. Each have decreased by 13.5, 82.5, 94.3, 64.8, 100, and 100%, respectively [7]. Autoclaving (128°C; 20 min) red kidney beans reduces tannin (42.9%) and phytic acid (48.61%) and increases the protein digestibility (45.3%) [9]. Boiling asparagus bean for 20 minutes significantly decreases phytate (48%), tannin (38%), and trypsin inhibitor (75%) [11].

There is scarce information in the literature about nutritional and antinutritional Kabau. Considering that Kabau is one of the original Indonesian commodities, it is essential to explore its nutritional, antinutritional, and protein digestibility characteristics and the effects of processing on them, so this research needs to be done. Thus, this study was designed to provide some analytical information on Kabau after conventional processing to increase Kabau protein's digestibility. This research is aimed at elucidating the effect of traditional processing (steaming, boiling, and frying) to change nutrition and antinutrition components and the *in vitro* protein digestibility of Kabau.

## 2. Materials and Methods

### 2.1. Sample Preparation

The fresh Kabau was harvested from a traditional yard in Bengkulu, Indonesia. The outer skin of Kabau was removed, and the peas were washed with tap water and then were drained. The fresh peas immediately were moisture content analyzed, then dried by a cabinet dryer (50°C; 18 hours). The other peas were processed with steam blanching, boiling water blanching, and frying.

### 2.2. Reagents and Standards

The solvents and chemical reagents used for the extraction and component analysis were analytical grades, such as petroleum ether, dimethyl sulphoxide, tris-buffer pH 8,2, HCL, acetic acid, amyl alcohol, H_2_SO_4_, 4% boric acid, sodium phytic (SIGMA-P8810), HNO_3_, FeCl_3_, HCl, pepsin (SIGMA-77161), trypsin (SIGMA-T6763), pancreatin (SIGMA-P1750), NaOH, buffer phosphate pH 8, Folin-Ciocalteu reagent (SIGMA-F9252), ammonium thiocyanate, trichloroacetic acid (TCA) (MERCK-100807), *α*-*N*-benzoyl-DL-arginine-*p*-nitroanilide hydrochloride (BAPNA) (SIGMA-B4875), and *O-phthalaldehyde* (OPA). Solvent and reagents used for amino acid profile analysis were HPLC grade, and they were purified through a Millipore filter (0.45 *μ*m). Mix amino acid was used to quantify amino acid profile as an external standard.

### 2.3. Methods

#### 2.3.1. Processing of Kabau

Fresh Kabau were subsequently shared into three equal parts and processed by steaming (100°C; 15 minutes), boiling (100°C; 6 minutes), and deep frying with vegetable oil (150°C; 3 minutes). The processed Kabau were then analyzed for moisture content and dried by a cabinet dryer (50°C; 18 hours), then grounded and sieved with a 40 mesh strainer. Powdered Kabau were then analyzed for its nutrition and antinutrition components including proximate analysis, amino acid profile, and IVPD, as well as phytic acid, tannin, and trypsin inhibitor.

#### 2.3.2. Determination of Nutritional Components

The nutritional components of peas were determined by AOAC procedures [12, 13]. Moisture, protein, lipid, ash, and carbohydrate contents were analyzed according to oven drying methods, the micro-Kjeldahl, Soxhlet extraction procedure, dry ashing method, and carbohydrate by difference [14], respectively.

#### 2.3.3. Amino Acid Profile Determination

*(1) Sample Preparation*. Amino acid profile was performed according to Dai et al. [15]. 250 mg samples were hydrolyzed with 10 ml 6 M HCl+0.02% phenol reagent at 105°C for 24 hours. Briefly, the solution was neutralized with 10 ml 6 M NaOH. The released amino acids were derived by using the OPA reagent.

*(2) Derivatisation Reagent Preparation*. The OPA reagent was prepared freshly. 50 mg OPA and 1.25 ml methanol were added to a dark bottle, followed by the addition of 11.2 ml sodium borate buffer (pH 9.5), 50 *μ*l 2-mercaptoethanol, and 0.4 ml Brij-35. The solution was mixed thoroughly, stored at 4°C, and used within 36 hours after preparation.

*(3) Mobile Phase Preparation*. Mobile phase A (sodium acetate 0.1 M, pH 7.2) was prepared by the addition of 27.3 g sodium acetate trihydrate to 1.6 l H_2_O on a glass bottle. 96 *μ*l HCl 6 M, 180 ml methanol, and 10 ml tetrahydrofuran were added. The solution was diluted to 2 l with H_2_O. Mobile phase B was 100% methanol and stored in a dark bottle.

*(4) HPLC Conditioning*. Amino acid profile analysis was determined by a HPLC system equipped with SCL-10 A VP, LC-10 AD VP controller system pump, and a fluorescence lab alliance TM 1200 series detector, with excitation and emission wavelengths of 340 nm at 455 nm, respectively (Shimadzu, Kyoto, Japan). The column was euro sphere C18 (4.6 × 250 mm, 5 *μ*m). Amino acids were separated with a gradient separation system and at a 1.1 ml/minute flow rate. The gradient elution scheme can be seen in Table 1.

#### 2.3.4. Determination of Antinutrition Components and In Vitro Protein Digestibility

*(1) Phytic Acid*. Phytic acid content was determined, according to Bulan and Sebayang [16]. A 0.1 g sample was extracted with 20 ml of HNO_3_ 0.5 M and shaken in a water bath shaker at 28-30°C for four hours. The extract was filtered using a filter paper No. 1. One ml of extract was added into 0.4 ml of distilled water, followed by 1 ml FeCl_3_ 0.005 M boiled for 20 min. The mixture was left to cool; then, 5 ml of amyl alcohol and 0.1 ml of ammonium thiocyanate 0.1 M were added. The solution was mixed and centrifuged at 3000 rpm. The top layer was subsequently subjected to a UV-vis spectrophotometer at 495 nm. Standard curves of phytic acid were prepared with 50, 100, 150, and 200 ppm Na-Fitat solutions diluted with HNO_3_. The rate of phytic acid is expressed in mg/g of dry matter.

*(2) Tannin*. Tannin content determination was referred to Chanwitheesuk et al. [17]. A total of 1.25 g of powdered sample was extracted with 250 ml of distilled water and heated over boiling water for 2 hours. The extract was left to cool and filtered with Whatman filter paper No. 1. 1 ml of extract was added to 0.5 ml of Folin-Ciocalteu reagent and 2 ml of Na_2_CO_3_, then mixed and incubated for 30 min at room temperature. The solution was subjected to UV-vis spectrophotometry at 748 nm. The standard curve was prepared using a standard tannic acid at a concentration of 0.00, 0.01, 0.02, 0.04, 0.06, 0.08, and 0.1 mg/ml prepared in the same manner.

*(3) Trypsin Inhibitor*. Measurement of trypsin inhibitor (TI) activity was performed based on Kakade et al. [18], which has been modified by Coscueta et al. [19] and Malomo et al. [20]. TI activity measurement was done by preparing TI extract, BAPNA solution as substrate, and trypsin solution up to the TI activity testing stage. *TI extracts* were prepared by mixing 1 g of a powdered sample with 50 ml 0.01 M NaOH, agitated for three hours at room temperature. The TI extract was subsequently centrifuged for 10 minutes at 3500 rpm. The filtrate was separated as TI extract to determine its activity. *BAPNA stock solution* was prepared by dissolving 40 mg of BAPNA in 1 ml of dimethyl sulphoxide (DMSO), then diluted to 100 ml with 0.05 M tris-buffer pH 8.2. The BAPNA solution was placed at 37°C until use. BAPNA solution was always prepared before the TI assay. *Trypsin stock solution* was prepared by dissolving 4 mg of trypsin in 200 ml of HCl 0.001 M. Trypsin solution was stored in ice water bath. Trypsin solution was always prepared before the TI assay.

Measurement of TI activity begins by preparing a control solution (BAPNA and trypsin solution only) and sample solution (BAPNA, trypsin solution, and sample). The control solution was prepared by adding 2 ml of distilled water and 5 ml of BAPNA into 2 ml of trypsin solution in a water bath of 37°C for 10 min. Then, one ml of 30% acetic acid was added to the mixture to stop trypsin's reaction. It is followed by centrifugation at 3500 rpm for 10 minutes. The absorbance was read by UV-vis spectrophotometry at 410 nm. The sample solution was prepared as in the control solution by adding trypsin and BAPNA solution into 2 ml of sample extract. The blank was prepared by mixing 1 ml of 30% acetic acid, 2 ml of trypsin solution, and 5 ml of BAPNA solution. TI can be expressed as trypsin unit inhibited (TUI)/mg sample as Equation 1:

(1) $$\begin{array}{c} \frac{\text{TIU}}{\text{mg}}\text{sample}=\frac{\left(A1-A2\right)x\left(100/\text{df}\right)}{w/\text{df}}, \end{array}$$

where *A*1 is the control absorbance, *A*2 is the sample absorbance, *w* is the weight of the initial sample, and df is the dilution factor (ml).

*(4) Determination of In Vitro Protein Digestibility*. Determination of *in vitro* protein digestibility (IVPD) was based on Almeida et al. [21]. 250 mg of sample and 250 *μ*l of distilled water (for the blank) were suspended in 15 ml 0.1 M HCl containing 1.5 mg/ml pepsin and incubated in a water bath shaker at 37°C for 3 hours. The hydrolysis was stopped with the addition of 7.5 ml NaOH 0.5 M. Moreover, the pancreatic digestion process was done by adding 10 ml 0.2 M phosphate buffer (pH 8) containing 10 mg pancreatin and 1 ml sodium azide 0.005 M to prevent microbial growth and incubated in a water bath shaker at 37°C overnight. The pancreatin hydrolysis was stopped with the addition of 1 ml of 10% TCA, followed by centrifugation at 4500 rpm for 20 min. The supernatant was collected, and the total protein content was analyzed by the Kjeldahl AOAC method [12]. The IVDP value was calculated according to Equation 2:

(2) $$\begin{array}{c} \%\text{IVDP}=\frac{A-B}{A}\times 100, \end{array}$$

where *A* is initial protein (mg) and *B* is supernatant protein (mg).

### 2.4. Data Analysis

This experiment has four units, fresh and processed sample (steamed, boiled, and fried). Every unit experiment was done in two experimental replications and three analytical replications. Furthermore, there were six raw data for each unit experiment. Data were tested with ANOVA (Analysis of Variance), and if the treatment had significant effect, post hoc testing was carried out with DMRT (Duncan's Multiple Range Test).

## 3. Results and Discussion

### 3.1. Nutritional Components of Kabau

The nutritional components of Kabau are shown in Table 2. The steaming and boiling processes significantly (*P* < 0.05) increase the moisture content. The highest moisture content was in the boiling process compared to that of the other treatments. The fresh Kabau contained 44.71% moisture content, while boiled moisture content and steamed moisture content were 58.36% and 47.11%, respectively.

Moisture content was increased as a result of the contact between the peas with boiling and steaming medium. These results agree with those reported by Aluko [22], Harijono et al. [23], and Sobukola et al. [24]. Blanching such as steaming and boiling disrupted the cytoplasmic and other membranes, promoting the loss of cell turgor and softening of peas surface, and finally became more permeable [24, 25]. This phenomenon made it easy for water and vapor to penetrate the peas; therefore, the moisture content was increased, driving out gases and other volatile compounds [24].

The frying process could reduce moisture content significantly (*P* < 0.05). Frying at a high temperature could increase the water temperature inside. The evaporation process during frying was carried out in two steps. It was initiated by the bubbling process of water on the pea's surface due to the rise of water temperature but had not reached the boiling point yet. After the temperature had reached the boiling point, the bubble was then evaporated into its vapor form [26].

The ash content of Kabau at different processing is shown in Table 2. Ash content of all processing techniques showed no significant (*P* < 0.05) difference. The results obtained for ash content of processed and fresh Kabau were different from several studies. In contrast, soaking and boiling of peas could significantly decrease the ash content as the mineral from inside the peas would diffuse into the water for immersion or boiling [27].

Fat content of fried Kabau had significantly (*P* < 0.05) higher value compared to others (see Table 2). The fresh, boiled, steamed, and fried Kabau had 0.947%, 0.665%, 0.490%, and 12.86% fat content. The frying method had the highest value of fat content caused by oil penetration during the frying process. According to Moreira et al. [28], when the product was removed from the frying process, the temperature would drop and so as the pressure. The pores of the peas became more flexible, resulting in easier oil penetration, which initially could be observed at the surface. The oil inside the peas was then contributing to the total fat content of the fried Kabau. Dana and Saguy [29] explained that during the frying process, the water of the material was evaporated and rapidly reduced water concentration at surface material, followed by crust formation and water evaporation.

Crude protein of steamed and boiled Kabau is significantly higher (*P* < 0.05) compared to fresh Kabau (Table 2). On the contrary, the frying process did not show any significant crude protein change compared to fresh Kabau. Fresh, boiled, steamed, and fried Kabau had 27.57%, 39.79%, 32.43%, and 24.64% crude protein content. According to Olanipekun et al. [30], a significant increase in protein content after boiling and steaming processes could be a result of hydrolysis of large molecular proteins in the food into smaller molecules as peptides or amino acids. Hydrolysis of proteins during cooking was directly proportional to the increase in protein digestibility, thereby increasing protein value in the material [2]. The study confirmed Wang et al. [27], who reported that cooking could increase protein content of peas. The increases of protein might be attributed to the loss of soluble solids during cooking [27].

Reduced protein content in the frying process could have resulted from the processing temperature, which reached 150°C. High temperature could induce new molecules' formation as amino acids. Then, those amino acids could react with other compounds, such as aldehyde, epoxide, and hydroxyl ketone. Maillard reaction also occurred during the frying process [31].

HPLC quantitatively determined amino acids, and the amino acid profile is shown in Table 3. Processing techniques significantly decrease amino acid of the peas. Steamed and boiled Kabau showed no significant different amino acid composition. However, both of them are considerably higher compared to fried Kabau. Generally, steaming and boiling could affect any water-soluble component, as amino acids [25]. Losses of them are mostly due to leaching or diffusion [25, 32] and thermal destruction [25].

The Maillard reaction presents during frying [25]. It contributed to lowering amino acids such as lysine, histidine, and methionine. The carbonyl, formed from lipid oxidation, could also react with asparagine and formed acrylamide. This phenomenon was lowering protein content [31]. A high temperature of frying caused thermal destruction for most amino acids [25].

All of the samples were rich in threonine and lysine as essential amino acids. They were high in aspartic and glutamic acids as nonessential amino acids. According to Dermiki et al. [33], aspartic and glutamic acids in the free form are considered to have an umami taste. Thus, based on the high concentrations of aspartic and glutamic acids, Kabau potentially is used as a source of umami from indigenous Indonesian commodities through further research on free amino acids. There are many hydrophobic amino acids such as leucine and glycine, tyrosine as an aromatic amino acid, and lysine which has a vast side chain. These amino acids' presence can develop bioactive peptides as a hypotensive and antioxidant agent [34].

Fresh Kabau contained the highest amount of carbohydrate and are significantly different (*P* < 0.05) compared to all but steamed one (Table 2). Carbohydrate content of fresh, steamed, boiled, and fried Kabau was 69.84%, 65.72%, 58.03%, and 62.67%, respectively. The reduction in the carbohydrate value of Kabau might be caused by the leaching process of free sugars from the peas to water and oil during the boiling and frying process [35].

### 3.2. Antinutritional Components and *In Vitro* Protein Digestibility of Kabau

Antinutritional components that were studied are phytic acid, tannin, and trypsin inhibitor. Phytic acid can decrease bioavailability of some nutritional elements such as proteins, minerals, and starch. The phosphor of phytic acid can bind them, which could lower their bioavailability. Phytic acid is widely found in plants, especially on pulses. Fresh Kabau contained 2.02 mg of phytic acid/g dry matter. Processing such as steaming, boiling, and frying could significantly (*P* < 0.05) reduce the amount of phytic acid in Kabau, because of its sensitivity to high temperatures [11]. The value of phytic acid after processing is observed in Table 4.

Steamed Kabau had a significantly lower amount of phytic acid compared to the fresh one. The steaming was able to reduce phytic acid by up to 53% (Table 4). However, compared to the boiling and frying processes, the reduction of phytic acid by steaming was meager. The boiling and frying could reduce up to 95 to 96% of phytic acid (Table 4). According to Rehman and Shah [9], some legumes' boiling process could decrease phytic acid by up to 24–35.1%. Boiling of vegetable peas for 40 minutes could reduce phytic acid by up to 47.9% [36]. High-temperature treatments could damage phytic acid by hydrolyzing and degrading into inositol hexaphosphate, composed of penta- and tetraphosphate [7].

Tannins belong to the polyphenol compounds, which have bitter taste and could bind and form a precipitate with protein or other organic compounds. It was known as an antinutritional component due to its negative effect [37]. Kabau contains tannin in a significant amount. It can be seen in Table 4. The boiling process reduced a significant amount of tannin in Kabau by up to 35% (0.002 to 0.0013 mg/g dry matter). However, the steaming and frying processes did not show significant differences when compared to a fresh one. Alonso et al. [8] explained that heat processing could degrade tannin, decreasing its reactivity and ability to form any precipitate complexes with another molecules. Heat processing also changes the structure of tannin qualitatively [7].

Trypsin inhibitor (TI) belongs to the protease inhibitor group. It is highly unstable at high temperatures, so that the heat processing is very potent to reduce the TI of Kabau significantly. The value of TI is shown in Table 4. The fresh Kabau had 0.2543 TIU/mg matter; after the steaming, boiling, and frying processes, the value dropped as much as 0.0181, 0.0274, and 0.0185 TUI/mg matter, respectively. Reduction of TI activity was found to be around 89.2-92.8%. Wang et al. [27] stated that steaming showed higher effectiveness to reduce the TI activity. Temperature and time of processing had a significant effect on TI activity. Other literature showed that heat processing reduces TI activity in kidney beans [8]. Heat processing could significantly reduce TI activity as high temperature could change the TI structure and dissolve the TI components into the processing media [38]. Heat processing could induce deamidation reaction, covalent bond breakage, such as hydrolysis of the peptide, and broken disulphide bond [7].

According to Doss et al. [39], boiling of lentils significantly reduces the number of trypsin inhibitors and tannins. Boiling with distilled water at 100°C at a ratio 1 : 10 (*w*/*v*) for 20 minutes significantly reduced tannin levels by up to 64% and trypsin inhibitor levels by up to 62%. Decreased levels of tannins and trypsin inhibitors also occurred in sweet lupine seeds and bitter lupine seeds. The boiling process was performed with distilled water 1 : 10 (*w*/*v*) at 100°C for 40 minutes and decreased trypsin inhibitors up to 60.8% in bitter lupine seeds and 66.6% in sweet lupine seeds. Tannin levels significantly reduced to 0.8% in bitter lupine seeds and 17.4% in sweet lupine seeds. However, there was an increase in phytic acid content by up to 8% in bitter lupine seed and 5.5% in sweet lupine seeds [40]. The steaming of soybean, winged bean, and lamtoro also decreased trypsin inhibitor activity, 86.22%, 97.09%, and 97.36%, respectively. These results were similar to the boiling process of soybean, winged bean, and lamtoro, which could also decrease trypsin inhibitors' activity. The decrease of trypsin inhibitor activity was 87.75%, 96.35%, and 99.14%, respectively [41].

Processing significantly increased *in vitro* protein digestibility of Kabau. The changes can be seen in Table 4. Boiling had the highest value of *in vitro* protein digestibility, as much as 69.47%, followed by steamed Kabau, as much as 61.48%. The frying processing had the lowest value, 43.64% lower than fresh Kabau. According to Table 4, fried Kabau contained the lowest total protein amount, even compared to the fresh one. This result of antinutritional compounds has led to the same thing occurring in *in vitro* protein digestibility.

Steaming and boiling could degrade some of antinutritional components; therefore, protein digestibility evaluation showed a high value. As described before, high-temperature processing reduced tannin and phytic acid so that more soluble protein could be hydrolyzed and increased its bioavailability [7, 8, 36]. Steaming and boiling also reduce trypsin inhibitor activity. Trypsin inhibitors can inhibit trypsin activity, which is characteristic of the human gastrointestinal tract. Trypsin inhibitors have very stable complexes with digestive enzymes, especially trypsin [42]. Thermal processing causes the breaking of intermolecular bonds responsible for holding the trypsin inhibitor's tertiary structure [43]. So, steaming and boiling could break the trypsin inhibitor structure down. Therefore, its activity could reduce, and protein bioavailability increases [7, 8, 36, 38]. In addition, thermal processing can cause structural changes in proteins, making them more accessible to proteases [40]. Moreover, increasing the protein digestibility may be related to the decreasing cell wall rigidity [40] and increasing cell permeability [44] during steaming and blanching.

## 4. Conclusions

Steaming did not show any significant differences compared to the fresh peas in regard to the nutritional components. Boiling increases protein and decreases the carbohydrate of Kabau. Both steaming and boiling Kabau can retain amino acid profile qualitatively, but they reduced amino acid concentration. Frying decreased protein content significantly and has the lowest value of almost all amino acids. All processing could substantially decrease the antinutritional components. The best processing technique to increase *in vitro* protein digestibility was the boiling process. The results of this study showed that the boiling process was the recommended method to process Kabau.

## Figures and Tables

**Table 1:** Gradient elution scheme for amino acid profile determination.

Time (minutes)	Solvent A (%)	Solvent B (%)
0	86	14
14	80	20
18	70	30
18.1	55	45
24	50	50
26	30	70
30	0	100
32.1-39	86	14

**Table 2:** The nutrition components of Kabau on different conventional processing techniques.

	Fresh	Steaming	Boiling	Frying
Moisture content (%wb)	44.71 ± 0.15^c^	47.11 ± 0.76^b^	58.36 ± 0.65^a^	39.32 ± 0.21^d^
Ash content (%db)	1.59 ± 0.08^a^	1.42 ± 0.04^a^	1.30 ± 0.17^a^	1.32 ± 0.19^a^
Fat content (%db)	0.94 ± 0.12^a^	0.49 ± 0.10^a^	0.66 ± 0.22^a^	12.86 ± 0.39^b^
Protein content (%db)	27.57 ± 0.83^c^	32.43 ± 1.35^b^	39.79 ± 2.05^a^	24.64 ± 0.89^c^
Carbohydrate content (%db)	69.84 ± 0.87^a^	65.72 ± 1.23^ab^	58.03 ± 2.12^c^	62.67 ± 0.67^b^

^∗^wb: wet basis; ^∗∗^db: dry basis. Data are presented as the mean ± standard error (*n* = 6). Mean values with different superscript letters in the same line indicate a significant difference (*P* < 0.05).

**Table 3:** Amino acid profile of Kabau on different conventional processing techniques.

Amino acid	Amino acid concentration of Kabau (mg/100 g protein DW)			
	Raw	Steam blanching	Boiling	Frying
*Essential amino acid*				
Arginine	4.44 ± 0.57^a^	2.18 ± 0.01^b^	2.46 ± 0.19^b^	0.61 ± 0.13^c^
Threonine	9.10 ± 0.06^a^	8.07 ± 0.01^b^	7.39 ± 0.01^c^	6.10 ± 0.007^d^
Methionine	0.89 ± 0.02^a^	0.75 ± 0.02^b^	0.70 ± 0.003^b^	0.59 ± 0.01^c^
Phenylalanine	4.42 ± 0.01^a^	2.76 ± 0.01^b^	2.78 ± 0.003^b^	0.03 ± 0.008^c^
Isoleucine	4.45 ± 0.004^a^	4.16 ± 0.07^b^	4.08 ± 0.07^b^	2.24 ± 0.01^c^
Leucine	7.75 ± 0.08^a^	6.28 ± 0.05^b^	5.79 ± 0.05^c^	5.38 ± 0.07^d^
Lysine	9.85 ± 0.08^a^	8.49 ± 0.41^b^	8.12 ± 0.03^bc^	7.45 ± 0.48^c^
Valine	4.29 ± 0.33^a^	2.79 ± 0.05^b^	2.98 ± 0.007^b^	0.94 ± 0.01^c^
Total	45.19	35.48	34.30	23.34
*Nonessential amino acid*				
Aspartic acid	11.32 ± 0.03^a^	7.43 ± 0.05^c^	9.11 ± 0.09^b^	5.50 ± 0.007^d^
Glutamic acid	17.27 ± 0.02^a^	15.74 ± 0.06^c^	16.75 ± 0.23^b^	12.39 ± 0.23^d^
Tyrosine	9.90 ± 0.08^a^	7.44 ± 0.03^c^	8.16 ± 0.006^b^	6.33 ± 0.004^d^
Glycine	6.34 ± 0.34^a^	5.25 ± 0.000^b^	5.04 ± 0.005^b^	4.97 ± 0.001^b^
Alanine	3.24 ± 0.28^a^	3.03 ± 0.02^a^	2.23 ± 0.02^b^	1.42 ± 0.02^c^
Serine	10.57 ± 0.02^a^	7.27 ± 0.001^c^	8.42 ± 0.03^b^	6.89 ± 0.01^d^
Histidine	4.17 ± 0.06^a^	2.65 ± 0.02^b^	2.73 ± 0.008^b^	2.23 ± 0.006^c^
Total	62.81	48.81	52.44	39.73
Total (essential amino acid and nonessential amino acid)	108.00	84.29	86.74	63.07

Data are presented as the mean ± standard error (*n* = 6). Mean values with different superscript letters in the same line indicate a significant difference (*P* < 0.05).

**Table 4:** Antinutritional components and *in vitro* protein digestibility of Kabau in different processing techniques.

Components	Fresh	Steaming	Boiling	Frying
Phytic acid (mg/g dry matter)	2.0270 ± 0.02^a^	0.9428 ± 0.04^b^	0.1024 ± 0.00^c^	0.0758 ± 0.00^c^
Tannin (mg/g dry matter)	0.0020 ± 0.00^a^	0.0016 ± 0.00^ab^	0.0013 ± 0.00^c^	0.0016 ± 0.00^ab^
Trypsin inhibitor (TUI/mg dry matter)^∗^	0.2543 ± 0.03^a^	0.0181 ± 0.00^b^	0.0274 ± 0.00^b^	0.0185 ± 0.00^b^
*In vitro* protein digestibility (%)	47.59 ± 1.05^c^	61.48 ± 0.69^b^	69.47 ± 0.96^a^	43.63 ± 0.63^d^

^∗^TUI: trypsin unit inhibited. Data are presented as the mean ± standard error (*n* = 6). Mean values with different superscript letters in the same line indicate a significant difference (*P* < 0.05).

## Data Availability

The data used to support the findings of this study are available from the corresponding author upon request.
